# Supplementary material for: Effects of miR-306 Perturbation on Life Parameters in the English Grain Aphid, Sitobion avenae (Homoptera: Aphididae)
Source: Int J Mol Sci. 2024 May 23;25(11):5680. doi: 10.3390/ijms25115680 (PMC11171923; doi:10.3390/ijms25115680)
Supplement: Supplementary file 1 [file ijms-25-05680-s001.zip › ijms-3005960-supplementary.pdf]

Supplementary materials

Table S1. Sequence information of miRNA agomir

Name	Sequences (5′-3′)
miRNA-306 agomir	UCAGGUACCAAGUGAUUUCUGA<br>AGAAAUCACUUGGUACCUGAUU
NC agomir	UUCUCCGAACGUGUCACGUTT<br>ACGUGACACGUUCGGAGAATT
